# New features of pytransform3d for robotics research: transformation graphs, uncertainty, and 3D visualization in Python

**DOI:** 10.3389/frobt.2026.1860457

**Published:** 2026-10-16

**Authors:** Alexander Fabisch, Mihaela Popescu

**Affiliations:** 1 German Research Center for Artificial Intelligence GmbH (DFKI), Robotics Innovation Center, Bremen, Germany; 2 Robotics Research Group, University of Bremen, Bremen, Germany

**Keywords:** kinematics, rigid transformation, special Euclidean group, state estimation, uncertainty propagation, visualization

## Abstract

Rigid transformations in three dimensions (3D) are fundamental tools in robotics, computer vision and graphics, and physics simulation. However, there are many pitfalls for practitioners: various alternative representations of rigid transformations and uncertainty, each with its own competing conventions, sometimes without clear documentation, ambiguities in representations, singularities, and difficulties in handling many transformations in complex systems. This article presents the new features of pytransform3d, a Python library for working with rigid transformations in 3D that helps tackle these difficulties. Specifically, we show applications of its new batch data processing functions, uncertainty propagation, and 3D visualization. Furthermore, we present applications of its transformation graph TransformManager, a tool inspired by tf of the Robot Operating System (ROS), which has a subclass for loading robot kinematics and a new subclass that handles time-varying transformations. In examples from imitation learning, kinematics, state estimation, and log data processing, we demonstrate these features and show that only a few lines of code are sufficient to solve these complex problems correctly. The explicit terminology of the library makes this code readable and reviewable. The library has 100% test coverage of its core features, has a broader feature set than comparable libraries, and handles batch processing of spatial data in fewer lines of code and faster than its alternatives.

## 1 Introduction

pytransform3d is a tool for handling transformations in robotics. Fabisch (2019) presents the core features of the library. In this article, we present the new features of pytransform3d for handling graphs of transformations, batch processing, visualization, and uncertainty quantification. pytransform3d specifically targets use cases such as debugging, prototype implementation, log analysis, and data processing, e.g., for data science and machine learning in robotics. It is a library for the programming language Python, which is widely used, has an advanced scientific ecosystem, does not need complicated build system integration, and is easy to learn. The design philosophy of pytransform3d is not to hide anything. There is no magic: no operator overloading, no classes to wrap raw data (rotations or transformations) other than NumPy arrays (Harris et al., 2020), and no layers of abstraction. It is as transparent to the user as possible.

The core functionality of pytransform3d consists of representing rotations and rigid transformations in 3D ( SO(3) , where SO is a special orthogonal group, and SE(3) , where SE is a special Euclidean group) in various formats and providing operations such as normalization, composition, inversion, interpolation, application to vectors, singularity detection, and disambiguation. Several related libraries implement subsets of pytransform3d’s features. The robotics toolbox for Python (Corke and Haviland, 2021) has a broader scope, including inverse kinematics, trajectory generation, or state estimation. In contrast to pytransform3d, its spatialmath package uses an object-oriented approach to encapsulate the underlying numerical data used to represent rigid transformations, which is not convenient for batch data processing in visualization or machine learning. Several of the conversions between the representations of SO(3) are implemented in transforms3d (Brett and Gohlke, 2009). The C++ libraries manif (Deray and Solà, 2020) and Sophus (Strasdat and Lovegrove, 2024) implement many features for handling SO(3) and SE(3) that pytransform3d provides, e.g., Lie algebra operations for state estimation. Similarly, there are specific libraries that implement quaternions, e.g., pyquaternion (Wynn, 2015) or rowan (Ramasubramani and Glotzer, 2018). SciPy (Virtanen et al., 2020) recently introduced the RigidTransform class to scipy.spatial.transform with an interface similar to the Rotation class. In RigidTransform, conversion operations between the representations of SE(3) were adopted from pytransform3d (Shambaugh, 2025). ROS tf (Foote, 2013) manages a graph of time-stamped transformations, which is similar to pytransform3d’s TransformManager and TemporalTransformManager.

For data science and machine learning workflows in robotics, it is often necessary to process large datasets, handle graphs of transformations (e.g., kinematic chains) and uncertainty, and visualize data in 3D. However, there is no library besides pytransform3d that seamlessly vectorizes conversions of and operations on batches of rigid transformations and offers all tooling for handling graphs of transformations, uncertainty, plotting, and visualization. Table 1 summarizes this comparison. pytransform3d combines the representations of SO(3) and SE(3) , conversions between these, and operations on them with tools to handle complex graphs of transformations, visualization, plotting, and uncertainty. The library does not implement any new methodology. Its strengths lie in the integration of existing methods in well-curated software with 100% test coverage of its core features and years of application in robotic contexts. Another advantage of pytransform3d is its lightweight implementation with only a few dependencies, which simplifies deployment in a wide variety of systems and applications. C++ libraries such as manif have to be compiled during installation, and ROS tf depends on the ROS ecosystem. The boilerplate code for using pytransform3d is limited to converting data to and from NumPy arrays. pytransform3d is widely used, as indicated by its usage metrics (as of 8 April 2026): it was downloaded 2.4 million times from PyPI, 250,000 times during the week between April 1 and April 8, and 84,000 times with conda and has 701 stars on GitHub. The library is written in Python only and is platform independent. The current version 3.15.0 of pytransform3d is tested with Python 3.9–3.13 with continuous integration. For all code excluding plotting and visualization, 100% test coverage is achieved. It is released under the New BSD/BSD 3-Clause License. The latest version will be archived with Zenodo as Fabisch (2025b). The source code is available at https://github.com/dfki-ric/pytransform3d.

**TABLE 1:** Feature comparison of Python libraries for 3D rigid body transformations.

	Representations		Integration			
Library	SO(3)	SE(3)	Uncertainty	Graph	Batch operations	Visualization
pytransform3d	6	5	✓	✓	✓	✓
spatialmath	4	4	✗	✗	◦ ^a^	✓
transforms3d	4	1	✗	✗	✗	✗
manif	3	2	◦ ^b^	✗	✗	✗
rowan	4	✗	✗	✗	✓	✗
SciPy	5	3	✗	✗	✓	✗
ROS tf	✗	✗	✗	✓	✗	✓^c^

^a^ For instance, a vectorized construction of SE(3) objects from batches of positions and quaternions is not supported.

^b^ Provides adjoints and Jacobians; features such as covariance composition must be implemented manually.

^c^ Visualization via RViz.

✓: supported, ◦ : partial support (see footnotes), and ✗: not supported. Numbers count supported representations.

The structure of this article is as follows. In Section 2, we introduce concepts related to rigid transformations (representations and notations), show how they are implemented in pytransform3d, and give practical advice on how to use the library effectively. Building on these foundations, we discuss the more advanced topics of handling graphs of transformations and composing uncertain transformations. We furthermore introduce the plotting and visualization features of the library that play a crucial role for practitioners. We demonstrate how pytransform3d can be used to solve challenging kinematic problems and to analyze and visualize data in Section 3.

## 2 Methods

The pytransform3d library provides tools to work with 3D transformations in Python. It integrates well with the scientific Python ecosystem with its core libraries NumPy (Harris et al., 2020), SciPy (Virtanen et al., 2020), and Matplotlib (Hunter, 2007). The library implements operations for common representations of transformations, conversions between those representations, vectorized versions of operations and conversions, clear documentation of conventions, functions to check for common pitfalls (e.g., singularities, ambiguities, and discontinuities), organization of complex graphs of transformations, operations for uncertain transformations, and visualization and plotting tools. In this section, we give a concise overview of the relevant concepts of rigid transformations in 3D and their implementation in the library.

### 2.1 Background

Before discussing the features of pytransform3d, we define the basic terms that we use throughout this article, following Waldron and Schmiedeler (2008). A position of a rigid body in 3D Euclidean space is expressed as a vector $p\in R^{3}$ . A translation is a displacement, in which points move along parallel lines by the same distance. An orientation of a rigid body in 3D Euclidean space is defined by a set of three orthogonal basis vectors. A rotation is a displacement, in which points move about a rotation axis through the origin of the reference frame (fixed point) along a circle by the same angle. A pose is a combination of positions and orientations. A (proper) rigid transformation is a combination of translation and rotation. A (coordinate reference) frame in 3D Euclidean space is defined by an origin (position) and three orthogonal basis vectors (orientation), and it is attached to a rigid body. The pose of a rigid body (i.e., of its frame) is always expressed with respect to another frame.

Notation is crucial to describe and understand physical quantities in 3D. We use the explicit approach suggested by Furgale (2014): xBCA , for a physical quantity x of frame C with respect to frame B expressed in frame A . For example, tBCA is a translation, or ωBCA is an orientation vector of C with respect to B measured in A . Hence, a position of point P with respect to a frame A can be written as _*A*_ p≔tAPA .

We use the matrix notation $M_{BA}$ to define a mapping from frame A to frame B that can be expressed as a matrix multiplication (e.g., rotation or transformation matrix) so that we can verify the multiplication order by reading from right to left, for instance, when we transform a point _*A*_ p by pB=MBAA_p . The second index of the matrix and the left index of the point have to match; otherwise, the order is incorrect. Hence, we can also use a transformation matrix $T_{BA}$ that defines a transformation from frame A to frame B to represent the pose of frame A in frame B . This is just a different interpretation of the same matrix and similar to the interpretation of a vector from A to $P{\;}_{A}t_{AP}$ as a point pA .

### 2.2 Representations in pytransform3d

Representing transformations in the 3D Cartesian space is difficult mainly because representing rotations is also difficult. Table 2 gives an overview of the representations and operations implemented in pytransform3d, and Figure 1 shows how the most important of these are connected. Each representation REPR is associated with functions for input validation (check_REPR), normalization (norm_REPR), random sampling (random_REPR), and conversion (REPR_from_OTHER_REPR). Furthermore, some representations have REPR_requires_renormalization to check if renormalization is required, REPR_double to generate the other representations of the same transformation if the representation has a double cover, REPR_near_singularity to check if an instance of REPR is close to singularity (e.g., gimbal_lock), and assert_REPR_equal to raise AssertionError in case of inequality, which is necessary for unit tests. In the following subsections, we introduce the representations of rotations and rigid transformations that we use in the application examples.

**TABLE 2:** Overview of representations for rotations/orientations and transformations/poses in 3D.

			Operations							Ambiguities	
Representation	Symbol	Set	Inversion	Application to vector	Concatenation	Interpolation	Represents derivatives	Normalization	Singularity	Angle periodicity	Double cover
Rotation matrix	R	SO(3)	$R^{T}$	✓	✓	✓^c^	✗	✓	✗	✗	✗
Unit quaternion	$q,\hat{q}$	$S^{3}$	$q^{*}$	✓	✓	✓^d^	✗	✓	✗	✗	✓
Euler angles	(α,β,γ)	$R^{3}$	✗^a^	✗	✗	✗	✗	✓^f^	✓	✓	✗
Rotation vector	ω	$R^{3}$	−ω	✗^b^	✗	✓^d,e^	✓	✓^f^	✓	✓	✗
Logarithm of rotation	$\left[\omega \right]$	so(3)	$-\left[\omega \right]$	✗	✗	✓^e^	✓	✗^f^	✓	✓	✗
Modified Rodrigues parameters	ψ	$R^{3}$	−ψ	✓	✓	✗	✗	✓^f^	✓	✓	✓
Transformation matrix	T	SE(3)	$T^{-1}$	✓	✓	✓^g^	✗	✓	✗	✗	✗
Unit dual quaternion	σ	$\{p+\epsilon q|p\in S^{3},$ q∈H∧‖p+ϵq‖=1}	$p^{*}+\epsilon q^{*}$	✓	✓	✓^g^	✗	✓	✗	✗	✓
Position and quaternion	(p,q)	$R^{3}\times S^{3}$	✗	✗	✗	✓^h^	✗	✓	✗	✗	✗
Exponential coordinates	Sθ	$R^{6}$	−Sθ	✗	✗	✓^e^	✓	✓^f^	✗	✓	✗
Logarithm of transformation	$\left[S\theta \right]$	se(3)	$-\left[S\theta \right]$	✗	✗	✓^e^	✓	✗^f^	✗	✓	✗

Remarks: ✗ does not indicate that an operation is impossible. All operations can be implemented by converting to another representation. The following cases need an explanation.

^a^ Inversion of Euler angles in convention ABC (e.g., XYZ) requires another convention CBA (e.g., ZYX), reversing the order of angles, and taking the negative of these.

^b^ Rotation of a vector by a rotation vector requires the computation of Rodrigues’ formula, which implicitly converts it to a rotation matrix.

Interpolation (with spherical linear interpolation, SLERP).

^c^ Interpolation with SLERP via conversion to exponential coordinates of rotation (rotation vector).

^d^ Directly supports SLERP.

Normalization.

^e^ SLERP via fractions of the representation; requires another representation for concatenation.

^f^ Not required for numerical reasons but to avoid ambiguities in representation due to angle periodicity. Angle periodicity means that the representation uses an angle, and adding 2nπ,n∈Z results in the same rotation.

^g^ ScLERP via conversion to exponential coordinates of transformation.

^h^ Only SLERP for orientation component.

**FIGURE 1:**
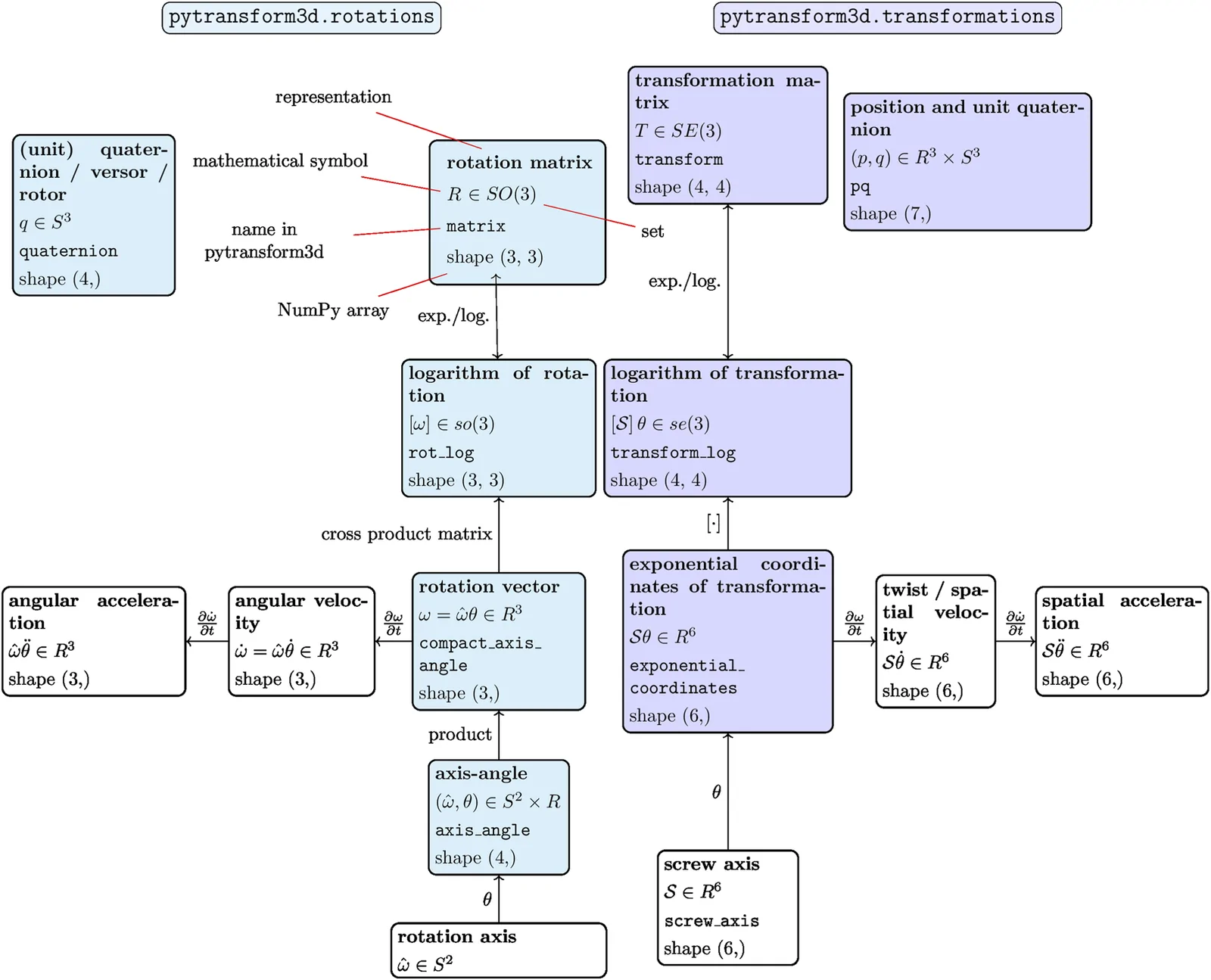
Overview of the selected representations of rotations and transformations in 3D and their implementation in pytransform3d.

#### 2.2.1 pytransform3d.rotations

The group of all rotations in the 3D Cartesian space is called SO(3) . The minimum number of components required to describe any rotation from SO(3) is 3. However, there is no minimal, continuous, and singularity-free representation. While the module pytransform3d.rotations implements the foundations to handle rotations, the module pytransform3d.batch_rotations implements vectorized operations on batches of rotations, e.g., conversions between representations.

##### 2.2.1.1 Rotation matrix

3×3 orthonormal matrices R∈SO(3) are a nonminimal representation of rotations with nine values but only three degrees of freedom and six constraints: the column vectors must have unit norm and must be orthogonal to each other, i.e., $R^{T}R=I$ (Lynch and Park, 2017). In addition, det(R)=1 because we use a right-handed coordinate system. With check_matrix(R), we can check all constraints. Hence, the group is defined as $SO\left(3\right)=\left\{R\in R^{3\times 3}\;|\;RR^{T}=I,\det\left(R\right)=1\right\}$ .

We can rotate a point pA from frame A to frame B with a rotation matrix $R_{BA}$ through the linear map:

pB=RBAA_pPython: B_p = R_BA @ A_p.

We concatenate multiple rotations by applying the rotation defined by $R_{CB}$ after the rotation $R_{BA}$ with the extrinsic convention through

$$R_{CA}=R_{C\underline{B}}R_{\underline{B}A}\;\left(\text{Python:}\;R\_CA\;=\;R\_CB\;@\;R\_BA\right).$$

Indices have to align to apply rotations in the correct order.

##### 2.2.1.2 Rotation vector (compact axis-angle and exponential coordinates of rotation)

Any rotation can be represented by a single rotation about one axis (see Figure 2). The axis can be represented as a 3D unit vector $\hat{\omega }\in S^{2}\subset R^{3}$ and the angle by a scalar θ . It is possible to write this as a more compact rotation vector: $\omega =\hat{\omega }\theta \in R^{3}$ (Waldron and Schmiedeler, 2008). We can represent angular velocity $\hat{\omega }\dot{\theta }$ and angular acceleration $\hat{\omega }\ddot{\theta }$ in this form.

**FIGURE 2:**
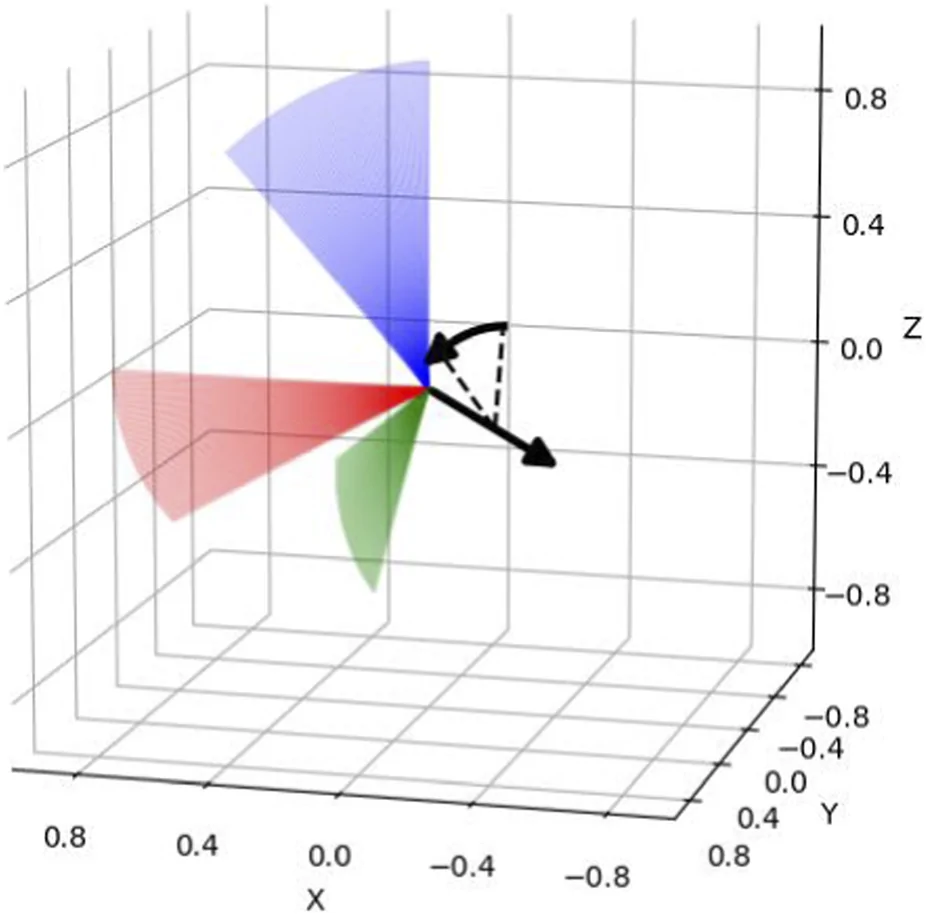
Rotation axis and angle applied to a reference frame. The straight black arrow is the rotation axis. The curved black arrow illustrates the rotation angle. The reference frame is represented by its red x-axis, green y-axis, and blue z-axis. We plot the reference frame at equally spaced intermediate steps of the application of the rotation operation. ☛ Example.

##### 2.2.1.3 Unit quaternion

Quaternions are represented by a scalar/real part w and an imaginary/vector part xi+yj+zk , i.e., q=w+xi+yj+zk∈H . Unit quaternions can be used to represent orientations with encoding based on the rotation axis and angle (Waldron and Schmiedeler, 2008). A rotation quaternion is a 4D unit vector (versor) $\hat{q}\in S^{3}$ and is based on the axis-angle notation through

$$\hat{q}=\left(\begin{array}{c} w\\ x\\ y\\ z \end{array}\right)=\left(\begin{array}{c} \cos\frac{\theta }{2}\\ \hat{\omega }\sin\frac{\theta }{2} \end{array}\right).$$

Unit quaternions are easy to renormalize by dividing by the vector norm. Quaternions can be written as a relatively compact, flat representation of SO(3) (a 1D NumPy array in comparison with a 2D NumPy array for a rotation matrix). Hence, they are often used to store orientations. However, there are several pitfalls related to quaternions:

- Hamilton vs. Shuster convention. There are two different quaternion products: Hamilton defines ijk=−1 , and Shuster defines ijk=1 . Sommer et al. (2018) explain why Hamilton’s convention should be used and give hints to identify which convention is used.
- Scalar first vs. scalar last. The scalar component w of a quaternion is sometimes the first element and sometimes the last element of the versor. We use the scalar-first convention, but this has to be checked when working with other systems or log data.
- Double cover. The *antipodal* unit quaternions $\hat{q}$ and $-\hat{q}$ represent the same rotation. This must be considered, e.g., in interpolation, distance calculation, or (approximate) equality checks.

We use the quaternion product $q_{CB}q_{BA}$ (Python: concatenate_quaternions(q_CB, q_BA)) to concatenate quaternions like we use the matrix product to concatenate rotation matrices. The inverse of the rotation q is its conjugate $q^{*}$ (Python: q_conj(q)). We rotate a vector by q by representing it as a pure quaternion v∈H (i.e., w=0 ) and using the quaternion product in $v^{\prime }=qvq^{*}$ (Python: q_prod_vector(q, v)).

#### 2.2.2 pytransform3d.transformations

The group of all proper rigid transformations (rototranslations) in the 3D Cartesian space is SE(3) . Transformations consist of a rotation and a translation and can be represented in different ways. While the module pytransform3d.transformations implements operations on individual rigid transformations, the module pytransform3d.trajectories provides functionality to handle trajectories, that is, sequences of poses.

##### 2.2.2.1 Transformation matrix

A convenient way of representing transformations are partitioned 4×4 matrices that contain a rotation matrix R∈SO(3) and a translation $t\in R^{3}$ of the form

$$T=\left(\begin{array}{cc} R & t\\ 0 & 1 \end{array}\right)\in SE\left(3\right),$$

which form the special Euclidean group SE(3) (Lynch and Park, 2017).

It is possible to transform position or direction vectors $v={\left(x,y,z\right)}^{T}$ through multiplication with it. Position vectors are represented as ${\left(x,y,z,1\right)}^{T}$ (Python: vector_to_point(v)), which activates the translation part of the transformation in a matrix multiplication. We deactivate the translation to transform a direction vector with ${\left(x,y,z,0\right)}^{T}$ (Python: vector_to_direction(v)).

##### 2.2.2.2 Notation conventions for composition of rigid transformations

We can use the following tricks to keep track of the right order for concatenation. When modeling transformations in mathematical equations, we often sequence them from right to left (extrinsic convention). Here, it makes sense to give names like $T_{BA}$ for transformations that map points from frame A to frame B , so we can see that $T_{CA}=T_{C\underline{B}}T_{\underline{B}A}$ when we read the names of the frames from right to left. Now, we can use the transformation matrix $T_{CA}$ to transform a point pA from frame A to frame C by multiplication pC=TCAA_p .

In Python, a convention that reads from left to right is more suitable to keep track of long sequences of transformations: A2C = concat(A2B, B2C). Similarly, we can transform a vector through p_in_C = transform(A2C, vector_to_point(p_in_A)). The notation is particularly effective when we use it in combination with longer, more readable frame names, e.g., left_hand_palm. Furthermore, pytransform3d provides functions to concatenate batches of transformations (in pytransform3d.trajectories): concat_many_to_one(A2Bs, B2C) composes a trajectory of frame A expressed in frame B with a transformation from frame B to frame C, concat_one_to_many(A2B, B2Cs) composes a transformation from A to B with a trajectory of frame B expressed in frame C, and concat_many_to_many(A2Bs, B2Cs) composes pairs of poses from two trajectories in efficient vectorized operations.

##### 2.2.2.3 Exponential coordinates of transformation

Just as any rotation can be expressed as a rotation by an angle about an axis, any transformation can be expressed by a motion along a screw axis (see, e.g., Lynch and Park, 2017; Corke, 2017). A screw motion translates along the screw axis and rotates about it. A screw axis is characterized by three **screw parameters** (see Figures 3, 4): a point $q\in R^{3}$ on the axis, the axis direction $\hat{s}\in S^{2}\subset R^{3}$ , and a pitch h∈R that encodes the ratio of translation to rotation. A **screw axis** is represented by

$$S=\left[\begin{array}{c} \omega \\ v \end{array}\right]\in R^{6},$$

where either ‖ω‖=1 or the screw axis represents only translation with ‖ω‖=0 and ‖v‖=1 . In the first case, we compute the screw axis from screw parameters $\left(q,\hat{s},h\right)$ as $\omega =\hat{s}$ and $v=q\times \hat{s}+h\hat{s}$ . In the second case, h is infinite, and we directly translate along $\hat{s}$ . By multiplication with another parameter θ , we can define a complete transformation through its exponential coordinates $S\theta \in R^{6}$ (sometimes the symbol τ is used). With only six values, this is a minimal representation. Twist (spatial velocity) can be expressed as $S\dot{\theta }$ .

**FIGURE 3:**
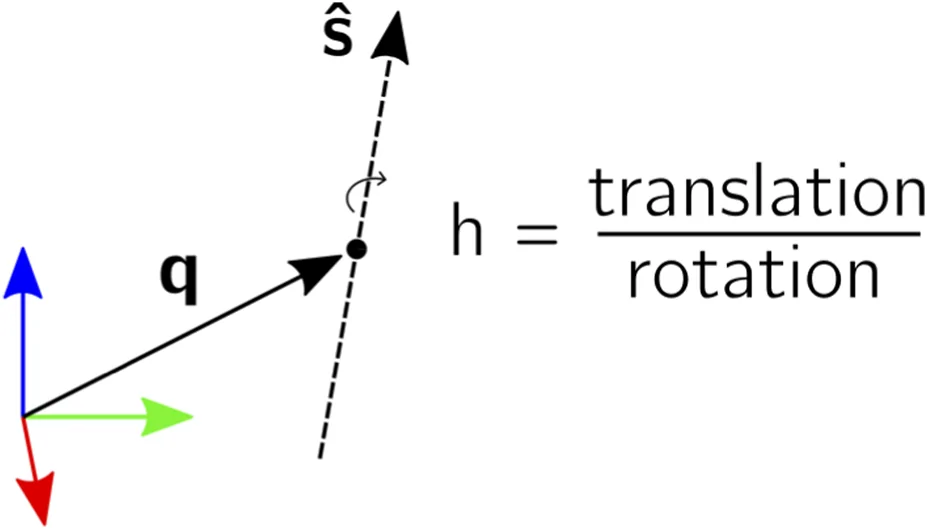
Parameters that define a screw axis. A screw axis is defined by the point q on the axis, a direction $\hat{s}$ , and the pitch h that defines the ratio between the translation and rotation.

We can use the exponential map $Exp:R^{6}\to SE\left(3\right)$ to compute a transformation matrix from exponential coordinates (Python: transform_from_exponential_coordinates(Stheta)) and the logarithmic map $Log:SE\left(3\right)\to R^{6}$ to compute the exponential coordinates from a transformation matrix (Python: exponential_coordinates_from_transform(A2B)).

### 2.3 Uncertainty: pytransform3d. uncertainty

We are almost never certain about transformations in robotics. pytransform3d.uncertainty provides tools to express uncertainty in rigid transformations. The module focuses on the geometry of the SE(3) manifold, namely, composition, inversion, and fusion operations of static uncertain transformations. The matrix exponential creates a nonlinearity in these operations.

We make the assumption of a Gaussian distribution with a mean represented by a transformation matrix and zero-mean Gaussian perturbations in the space of exponential coordinates that are translated to modifications of this mean transformation matrix. This allows us to model interdependencies between translation and rotation components through a covariance matrix over exponential coordinates. More specifically, we model uncertain transformations as normal distributions, where the mean is a transformation matrix T∈SE(3) and the covariance $\Sigma \in R^{6\times 6}$ is defined via exponential coordinates and the exponential map $Exp:R^{6}\to SE\left(3\right)$ ; that is, we assume that the mean is perturbed by random noise Exp(ξ)∈SE(3) , with ξ∼N(0,Σ) defined in exponential coordinates.

It makes a difference whether uncertainty is defined in a global frame of reference (i.e., left multiplied) or local frame of reference (i.e., right multiplied). For instance, to define uncertainty in a global frame, we assume that we sample from a Gaussian distribution of transformations $T_{xA}∼N\left(T|T_{BA},\Sigma_{6\times 6}\right)$ by computing $\Delta T_{xB}T_{BA}$ , with $\Delta T_{xB}=Exp\left(\xi \right)$ and $\xi ∼N\left(0_{6},\Sigma_{6\times 6}\right)$ (Barfoot and Furgale, 2014). Hence, the uncertainty is defined in the global frame B, not in the local body frame A. Vice versa, with $T_{BA}Exp\left(\xi \right),\;\xi ∼N\left(0_{6},\Sigma_{6\times 6}\right)$ , the uncertainty is defined in the local body frame A (Meyer et al., 2022).

Following Barfoot and Furgale (2014) and Meyer et al. (2022), pytransform3d.uncertainty implements operations such as composition, inversion, and fusion of uncertain transformations. We furthermore provide functions to visualize the equiprobable projected 6D hyperellipsoid at kσ intervals of a pose’s distribution in 3D.

### 2.4 A graph of transformations: pytransform3d.transform_manager

Managing all the transformations that are required to calculate new transformations is tedious. However, we can organize transformations in a graph like the one shown in Figure 5. Each frame is associated with a vertex and each transformation with an edge. Edges in these graphs are directed, but we can create an edge in the opposite direction by inverting the transformation. If we want to compute a new edge indicated by the dashed red arrow, we have to combine known transformations as indicated by the solid red arrow; that is, we compute the shortest path through the undirected graph, concatenate transformations along the path, and invert transformations if required. The TransformManager class of pytransform3d implements this. It is a lightweight version of ROS tf (Foote, 2013). The method add_frame(“A,” “B,” A2B) adds a transformation to the graph, and get_frame(“A,” “B”) requests a transformation with a minimum number of composition operations.

**FIGURE 4:**
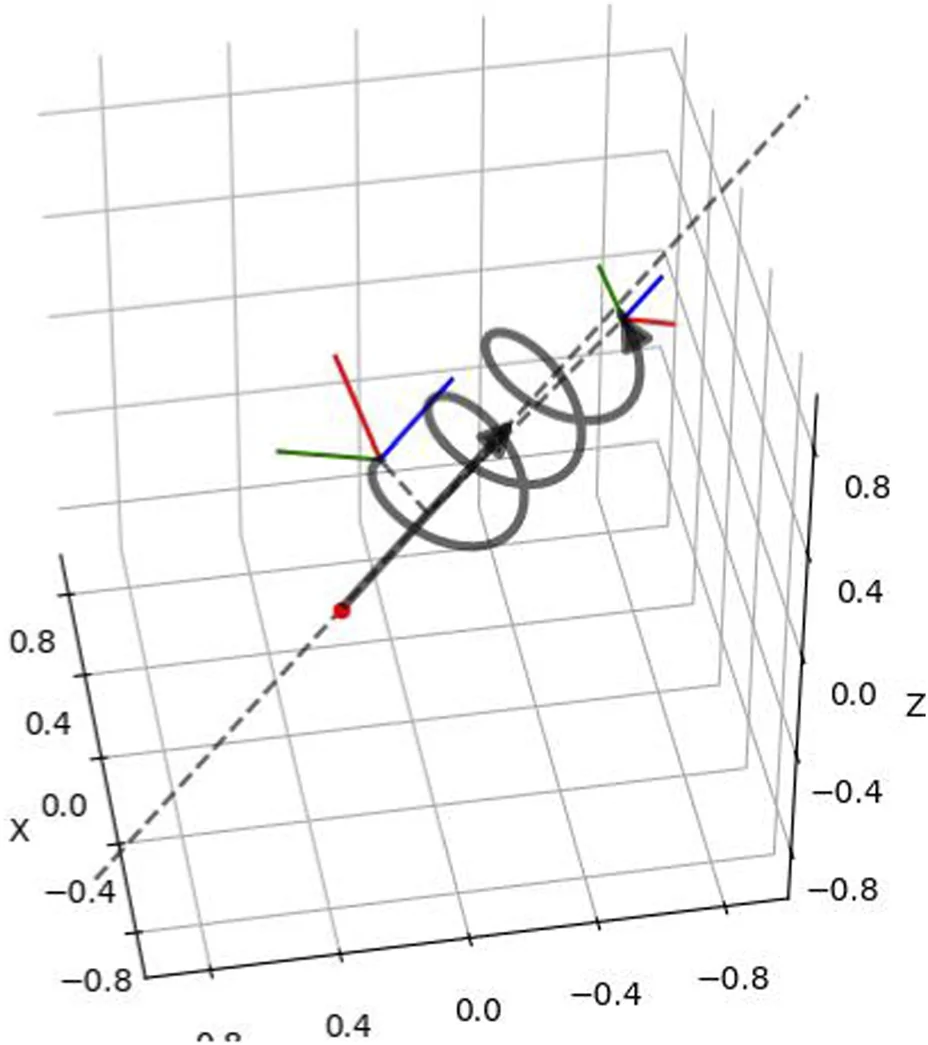
Illustration of a screw displacement operation. We can represent any transformation with a screw axis defined by parameters $\left(q,\hat{s},h\right)$ and another parameter θ that encodes the magnitude of the displacement. We visualize a screw axis and the transformation generated from a specific θ . The larger frame represents the origin of the transformation, and the smaller frame shows the transformed frame. A red point marks q , which is a point on the screw axis, and a straight arrow shows the direction $\hat{s}$ . The spiral path represents a displacement of length θ along the screw axis. ☛ Example.

**FIGURE 5:**
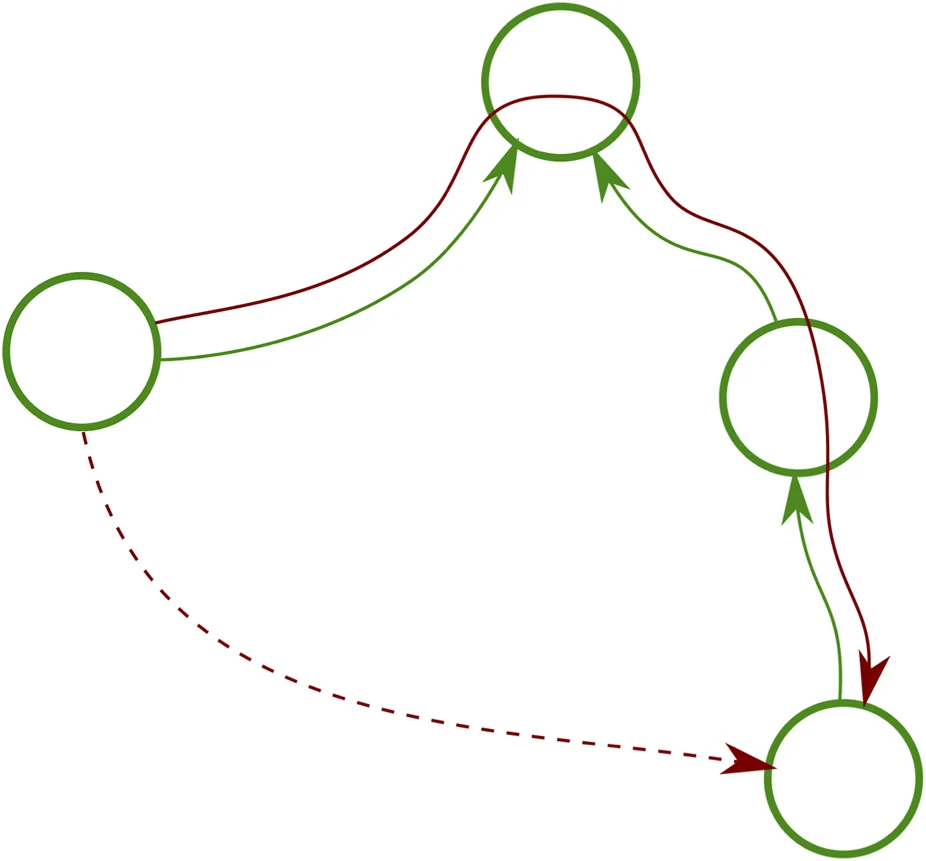
Example of a graph representing transformations that connect frames. Each node (green circle) in the graph is a frame. Edges (green arrows) of the graph represent known rigid transformations between these frames. When we need an unknown transformation (red dashed arrow), we can often compute it from known transformations (red solid arrow).

There are two variations of TransformManager. TemporalTransformManager handles time-varying transformations. UrdfTransformManager can load robot definitions from the unified robot description format (URDF) (ROS Developers, 2024) and display collision objects or visuals, which are spheres, boxes, cylinders, or meshes according to the URDF schema (see Figure 6a and d for examples). URDFs further define joints, which can be configured by the method set_joint(“joint_name,” joint_angle). Hence, a combination of setting joints and requesting a transformation between the end-effector frame and the base frame is a simple implementation of forward kinematics.

**FIGURE 6:**
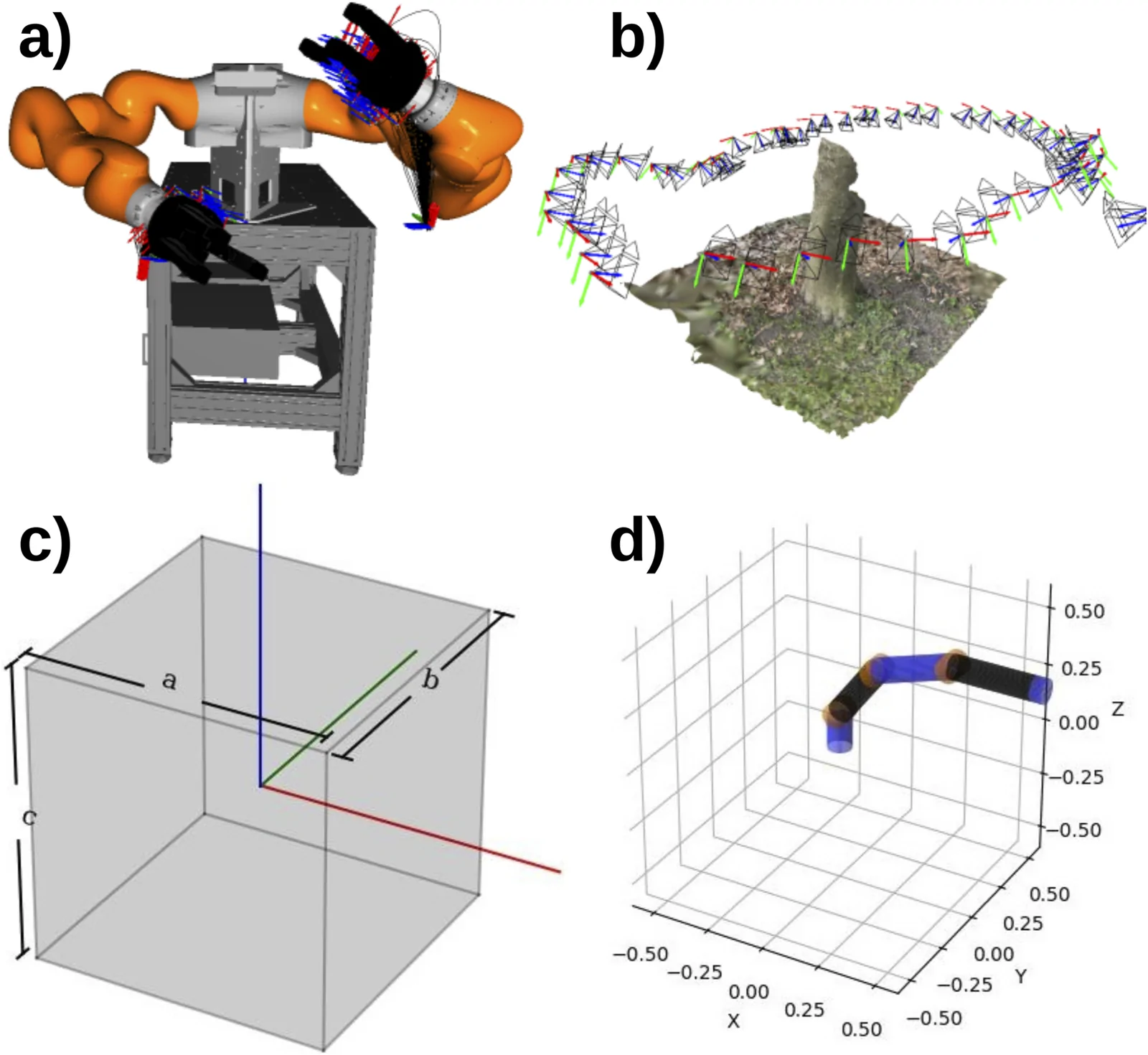
Examples of visualizations in Open3D and plots in Matplotlib implemented with pytransform3d. **(a)** Dual-arm robot and end-effector trajectories in Open3D. **(b)** Mesh from photogrammetry and corresponding estimated camera poses in Open3D. **(c)** Box with dimensions in Matplotlib. ☛ Example. **(d)** Robot arm with six joints in Matplotlib. ☛ Example.

### 2.5 Visualization and plotting

Visualization is essential to understand, debug, and communicate spatial information. pytransform3d is tightly coupled with Matplotlib (Hunter, 2007) and Open3D (Zhou et al., 2018) for visualization, plotting, and animation of 3D data (see Figure 6 for examples). We extend Matplotlib’s 3D plotting features to illustrate frames, arrows, cameras, graphs of transformations, URDFs, primitive geometries (e.g., spheres, cylinders, and boxes), and arbitrary meshes. The Open3D visualizer can be used for animations and static 3D visualizations. It not only has a high-level interface that resembles Matplotlib’s interface but also supports the extended set of visualization functions that we add to Matplotlib.

## 3 Results

Building on the foundations of Section 2, we demonstrate the features of pytransform3d by showing common use cases and replicating experiments from the robotics literature. Our goal is to qualitatively analyze how easy it is to translate kinematic and spatial data processing algorithms and concepts to Python code with pytransform3d. For this purpose, we specifically present one example of imitation learning that uses UrdfTransformManager and the Open3D visualizer; two examples from the fields of state estimation and robot kinematics that use methods for composition of uncertain rigid transformations and visualization features for uncertainty; and one example of log data analysis that uses TransformManager, batch conversion operations, and plotting of uncertainty and robot models.

### 3.1 Graph of transformations: automatic embodiment mapping

Transferring human hand motion recorded by a motion capture system to a robotic hand is a so-called automatic retargeting or embodiment mapping problem. Fabisch et al. (2022) propose using multichain inverse kinematics solvers to find a configuration of the MANO hand model (Romero et al., 2017) that closely matches the human hand configuration measured with a marker-based motion capture system. They use a second multichain inverse kinematics solver to find a configuration of a robotic hand that closely resembles the estimated configuration of the MANO model. These solvers match not only the fingertips but also the positions of previous finger segments. More specifically, the study transfers manipulation movements to two five-fingered robotic hands with 4 and 24 degrees of freedom. In this work, implementing multichain kinematics requires handling a complex graph of varying transformations loaded from a URDF description of the robotic hand, which is substantially facilitated with pytransform3d, and we focus on this aspect. However, pytransform3d was also used for visualization of robotic hands and manipulated objects.

UrdfTransformManager from pytransform3d.urdf was modified to compute forward kinematics faster with Numba (Lam et al., 2015) in FastUrdfTransformManager and then used to compute inverse kinematics of individual fingers of the hand simultaneously in MultiChain. Listing 1 highlights the most crucial parts of this implementation. Figure 7 shows optimization results for various hands and manipulation tasks with pytransform3d’s visualizer. The multichain inverse kinematics solver runs with a mean frequency of 128–181 Hz for the complex hand and 228–294 Hz for the simple hand, depending on the complexity of the movement. These frequencies can be reached even though the optimizer computes numerical gradients of the position errors, considerable parts of the code are implemented in pure Python, and the measurements were performed using a standard desktop computer. For a real-time application, the solver could be refined with exact gradients, an optimized C++ implementation, and parallelization of the forward kinematics, but for a prototype code like this, pytransform3d is well suited.

**FIGURE 7:**
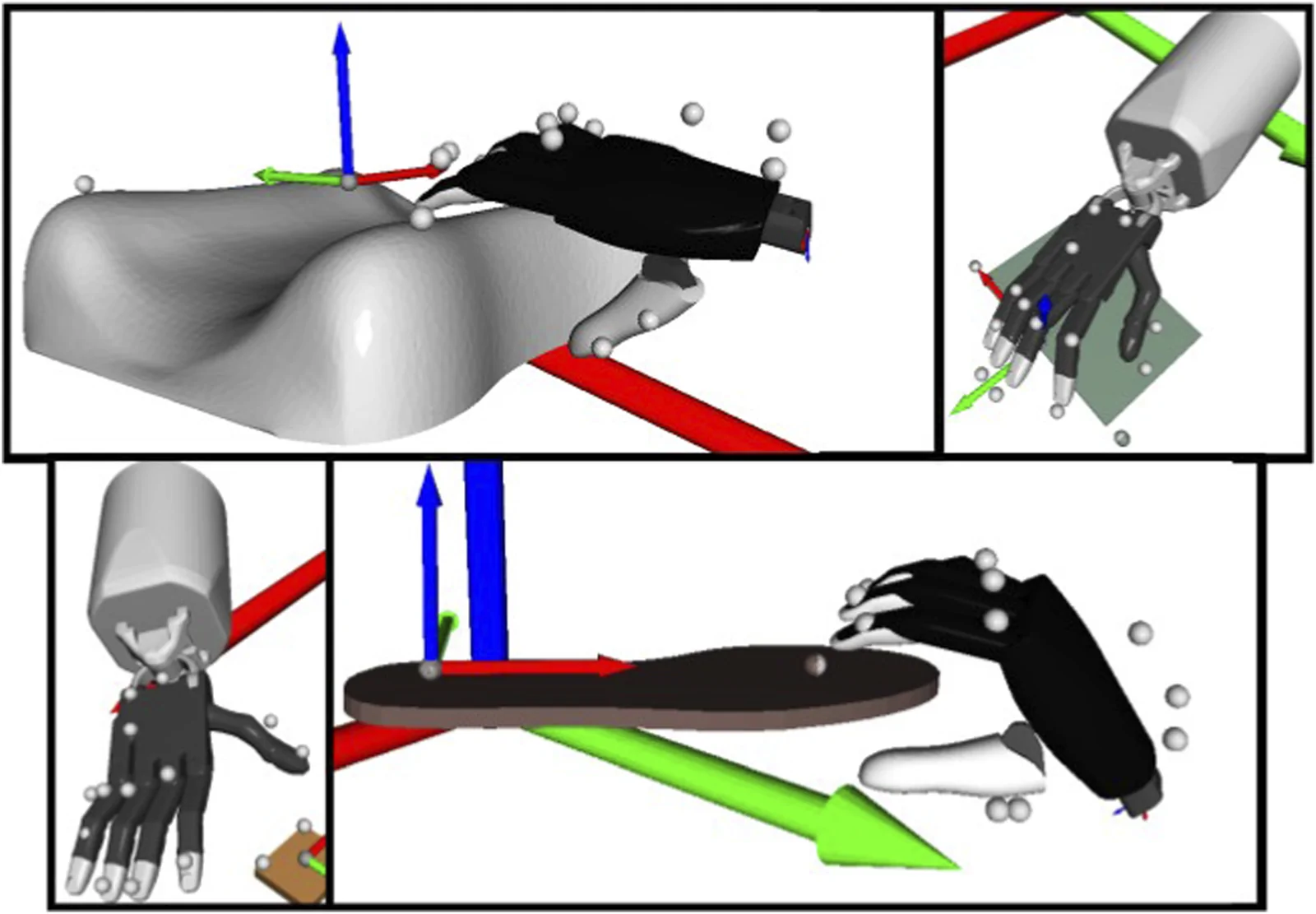
Automatic hand embodiment (Fabisch et al., 2022). Human motion capture data are translated to motions of robotic hands. Spheres represent measured positions of motion capture markers. The world frame (large coordinate system) and the object frame (small coordinate system) are displayed. In addition, a mesh of the manipulated object and the closest approximation of the human hand’s configuration by the robotic hand are displayed. ☛ Code.

Listing 1 Multichain inverse kinematics for a robotic hand. ☛ Code.

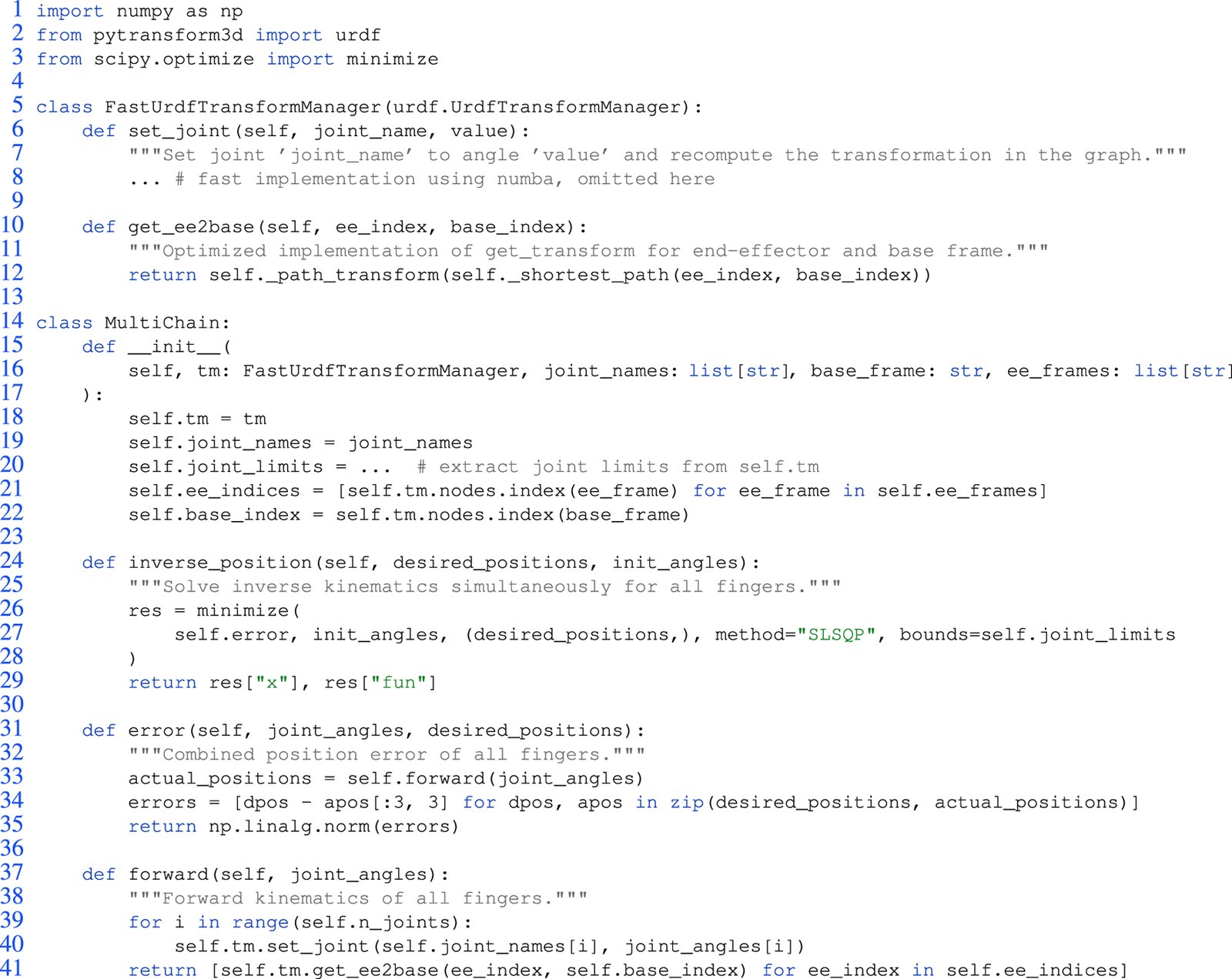

### 3.2 Uncertainty: composition of uncertain rigid transformations

The two examples in this section demonstrate composition of uncertain poses. While the state estimation example defines uncertainty in the global world frame (as proposed by Barfoot and Furgale (2014)), the example with robot arm kinematics defines uncertainty in the local body frame (as proposed by Meyer et al. (2022)).

#### 3.2.1 State estimation and banana distribution

This example is adapted from Barfoot and Furgale (2014). It computes uncertainty in the global reference frame. We assume a robot is moving at a constant velocity along the x-axis with noise in the angular velocity. Accumulation of noise leads to the so-called banana distribution of the final poses, which does not seem Gaussian in the Cartesian space, but it is in exponential coordinates (Long et al., 2013). Both the uncertainty propagation through state transitions and the visualization of uncertainty are implemented with pytransform3d (see Listing 2).

Listing 2 Uncertainty propagation on SE(3) with the resulting banana distribution. ☛ Example.

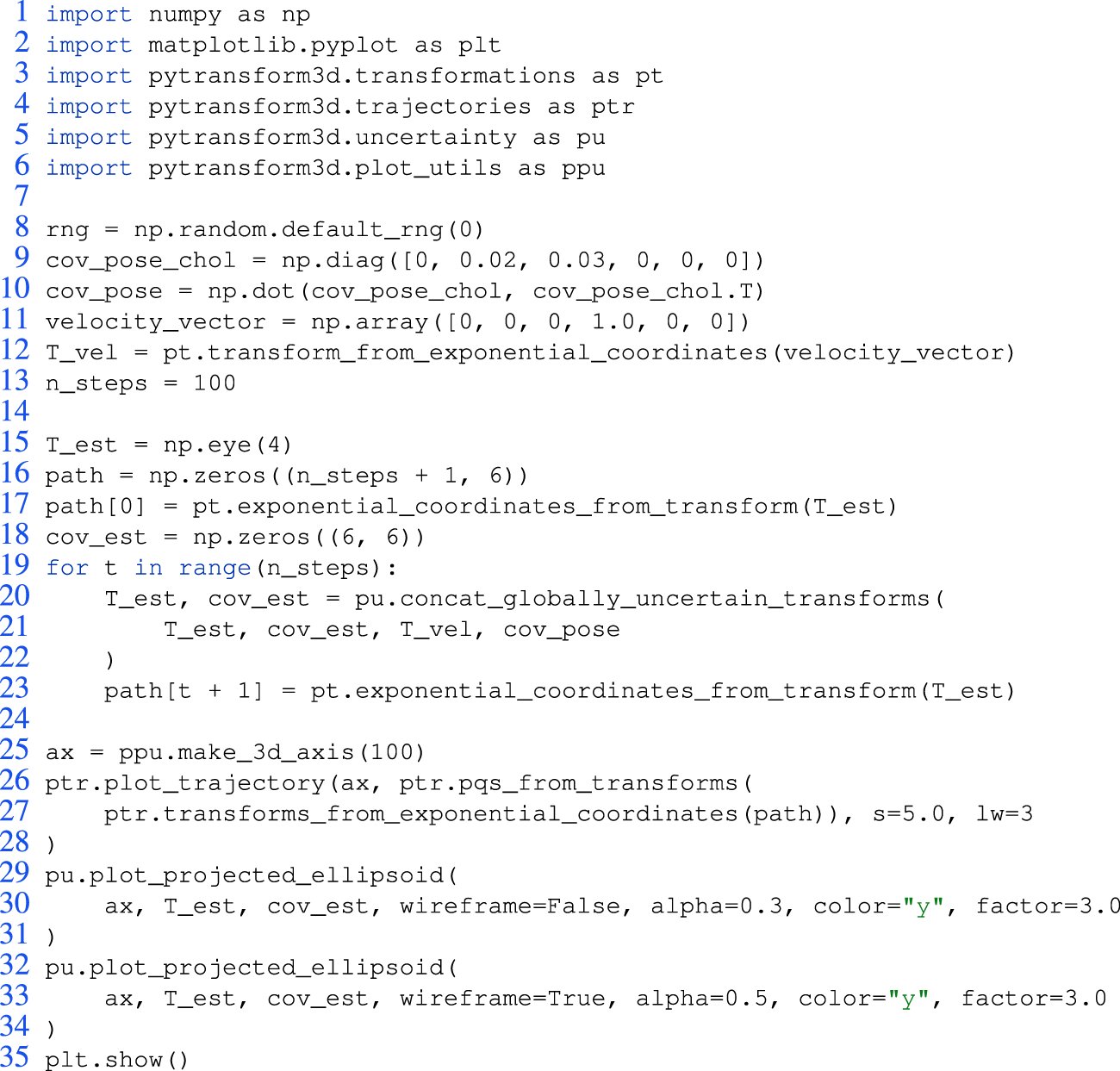

In each step, we concatenate $T_{\mathrm{vel}}=Exp\left(\Delta t\dot{\tau }\right)\in SE\left(3\right)$ defined via its exponential coordinates $\Delta t\dot{\tau }\in R^{6}$ , with Δt=1s . The covariance associated with $T_{\mathrm{vel}}$ is constructed from its Cholesky decomposition through ${LL}^{T}$ . Since it is a diagonal matrix, we effectively define the standard deviation of each velocity component. We introduce uncertainty in the rotational components of the velocity.

We compute the path with respect to the initial pose of the robot, which is $I_{4\times 4}\in SE\left(3\right)$ . The initial covariance of the pose is $0_{6\times 6}$ . In each step, we concatenate the current pose with $T_{\mathrm{vel}}$ and combine the covariance of pose and transformation with concat_globally_uncertain_transforms(A2B, cov_A2B, B2C, cov_B2C), which implements the composition operation derived by Barfoot and Furgale (2014). Hence, uncertainty accumulates in the covariance of the pose. For comparison, we perform Monte Carlo sampling of the trajectories but omit the code here for brevity.

A visual representation of the Gaussian distributions in 3D is an equiprobable ellipsoid. Equiprobable ellipsoids are equivalent to the $k\sigma ,k\in R^{+}$ intervals of a one-dimensional (1D) Gaussian distribution. However, transformations also have interaction between rotation and translation components so that an ellipsoid is not an appropriate representation to visualize the distribution of transformations in 3D. We must project a 6D hyperellipsoid to 3D, which can result in banana-like shapes (see Figure 8). This shape could be derived from Monte Carlo simulations, but it also emerges from the covariance over exponential coordinates of transformations (Long et al., 2013).

**FIGURE 8:**
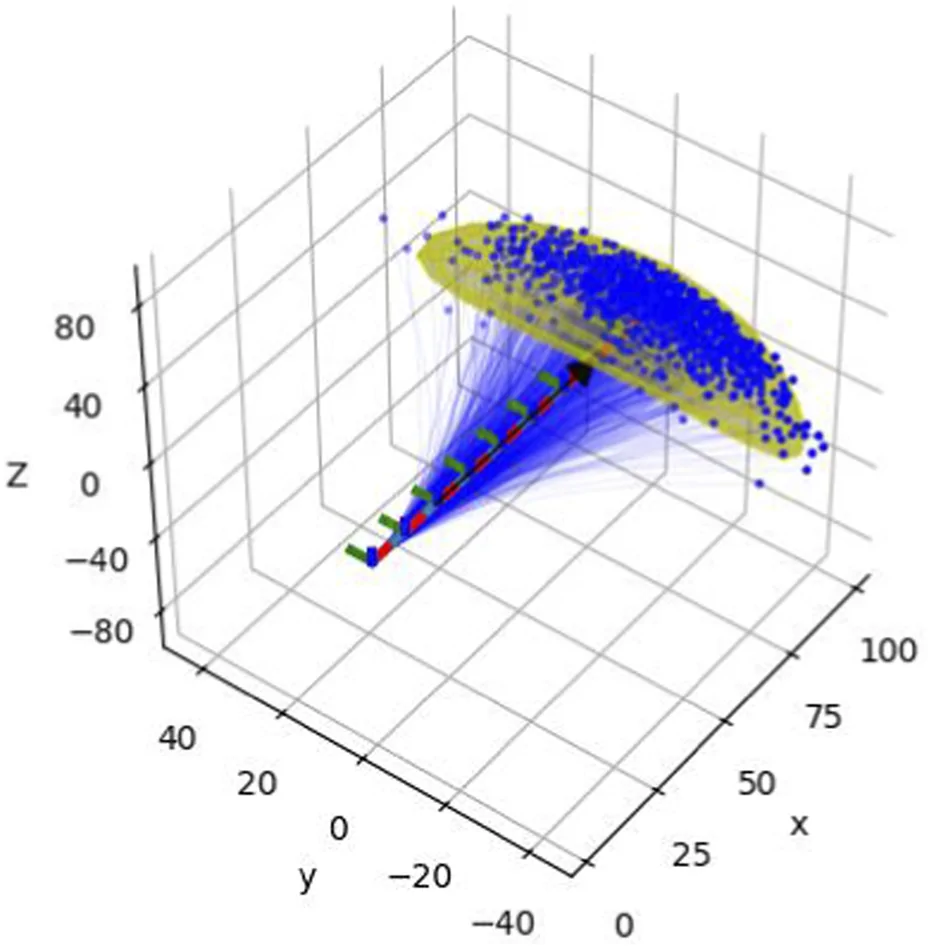
Banana distribution. The yellow mesh is the 3σ confidence volume of the final pose. ☛ Example.

#### 3.2.2 Probabilistic product of exponentials

Following Meyer et al. (2022), we implement the probabilistic product of exponentials (PPOE) for probabilistic forward kinematics of a robotic arm. PPOE uses a definition of uncertainty in the local reference frame, which is implemented by pytransform3d. The robot’s description is loaded by a subclass of UrdfTransformManager.

We omit the part of this example that converts the kinematics parameters from URDF to screw axes that are needed for the product of exponentials formulation of forward kinematics as this is lengthy and not conceptually complex. Based on these screw axes, the implementation of PPOE (Meyer et al., 2022) in the class ProbabilisticRobotKinematics (subclass of UrdfTransformManager) is as follows:

1. We multiply the screw axis of each joint with the corresponding joint angle to obtain the exponential coordinates of each relative joint displacement.
2. We concatenate the relative joint displacements and the base pose to obtain the end-effector’s pose. This is the original product of exponentials (Lynch and Park, 2017).
3. The PPOE modifies the original product of exponentials by transforming and concatenating the covariances of each transformation.

With pytransform3d, we can use the function concat_locally_uncertain_transforms(A2B, B2C, cov_A, cov_B) to concatenate joint displacements and propagate uncertainty (see Listing 3). In the end, we transfer the covariance from the end-effector frame to the base frame with the adjoint that we obtain with adjoint_from_transform(A2B). To visualize the 6D covariance, we project its equiprobable hyperellipsoid to 3D and visualize it as a mesh (see Figure 9) similar to the previous example.

Listing 3 Probabilistic product of exponentials. ☛Example.

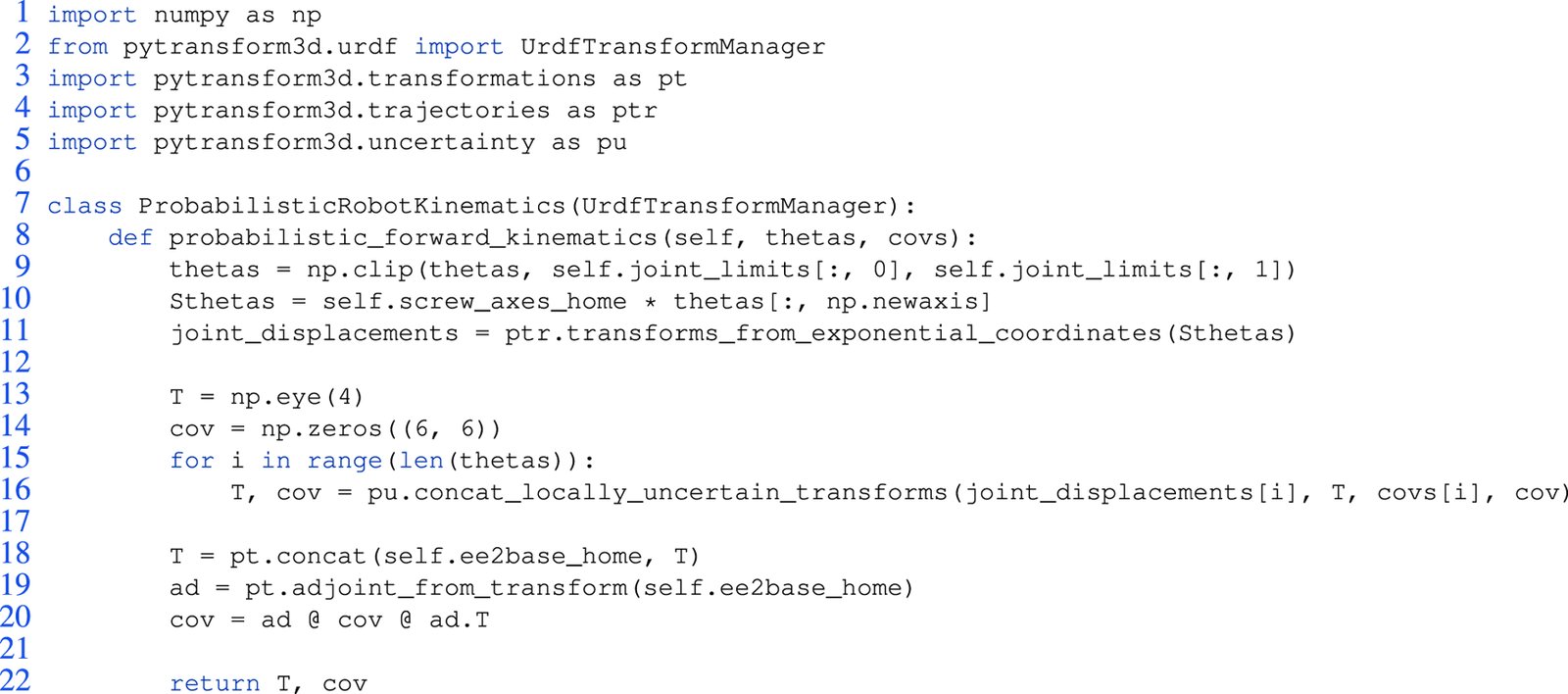

**FIGURE 9:**
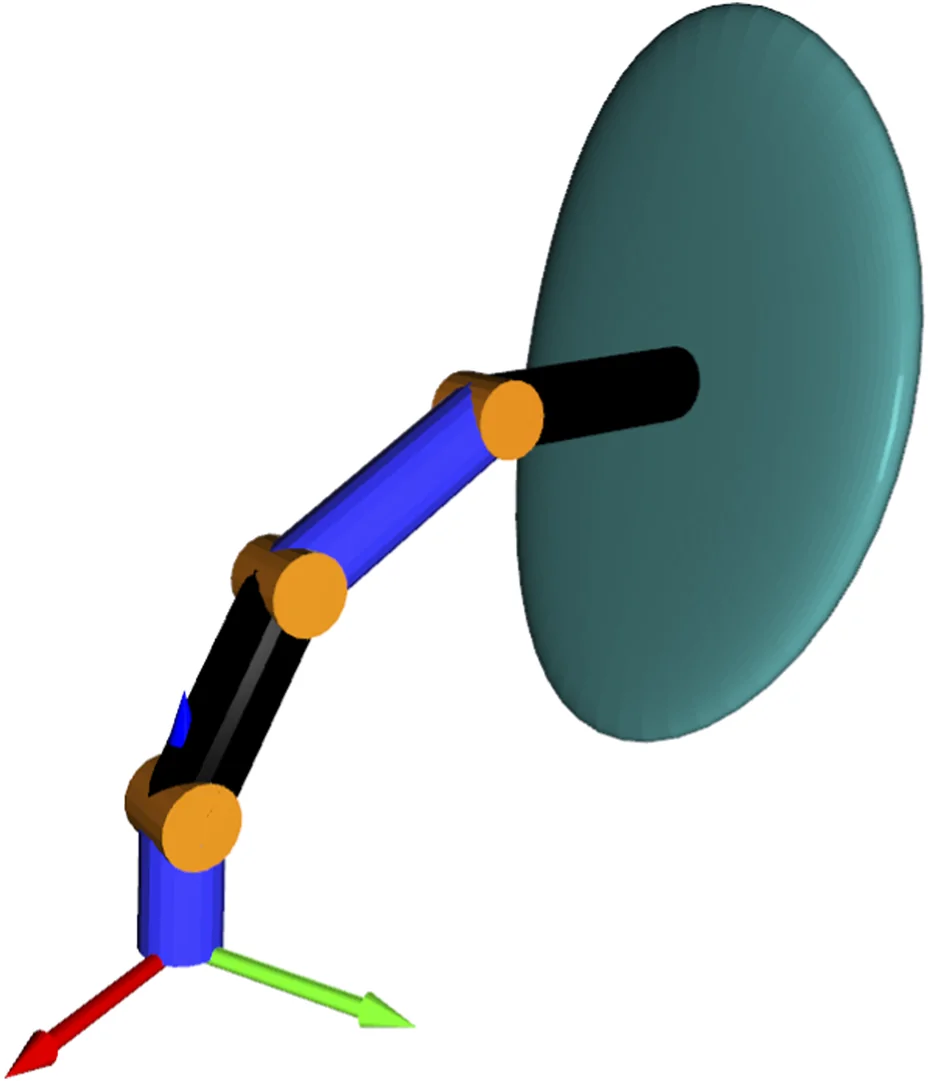
Probabilistic product of exponentials. The green mesh is the σ confidence volume of the end-effector pose. ☛ Example.

### 3.3 Plotting: illustration of quadruped state estimation on field test data

In the context of legged robots that traverse challenging terrain, floating base state estimation provides feedback to the whole-body controller to ensure stable and robust locomotion. To evaluate the accuracy of state estimation on unstructured and rocky terrain, Popescu et al. (2026a) conducted locomotion experiments on Vulcano Island using the Unitree Go2 quadruped robot, of which the data are publicly available (Popescu et al., 2026b). In this example, we use pytransform3d for log data analysis, including batch processing of estimated states and plotting the robot state and uncertainty.

The dataset (Popescu et al., 2026b) includes the inertial measurement unit (IMU) and joint encoder data at 1,000 Hz that are used for state estimation, as well as ground truth data from a real-time kinematic (RTK)/global navigation satellite system (GNSS) sensor providing accurate centimeter-level positioning at 10 Hz. Popescu et al. (2026a) perform state estimation by fusing IMU measurements with leg kinematics during ground contact with an invariant extended Kalman filter (InEKF) (Hartley et al., 2020). The robot state X is an element of the matrix Lie group $SE_{N+2}\left(3\right)$ , an extension of SE(3) that augments the pose (of the floating base) with velocity $v\in R^{3}$ and N contact positions $p_{c_{i}}\in R^{3}$ between legs and the ground. The filter estimates uncertainty of the state X with the state covariance matrix P . While the state matrix X is defined on the Lie group $SE_{N+2}\left(3\right)$ , the prediction and update steps are performed in the Lie algebra $se_{N+2}\left(3\right)$ ; that is, the state error and the associated covariance matrix P are defined in $se_{N+2}\left(3\right)$ . To analyze uncertainties of the pose, we extract its marginal distribution by removing the rows and columns of P that are not (co)variances of two pose components to obtain a covariance matrix $\Sigma \in R^{6\times 6}$ in se(3) . In the left-invariant formulation of the InEKF, the covariance matrix is expressed in the body frame {B}. To transform the uncertainty to the world frame {W}, we apply the adjoint transformation $Ad_{T_{WB}}$ , which maps tangent vectors from the body frame to the world frame. The transformed covariance is given by $\Sigma_{W}=Ad_{T_{WB}}\Sigma_{B}Ad_{T_{WB}}^{T}$ .

Algebraically, the state error in se(3) represents perturbations of orientations and positions, i.e., angular and linear velocities. Geometrically, this corresponds to the twist $S\dot{\theta }$ in screw theory (see Section 2.2.2.3). The covariance matrix P thus captures uncertainty in se(3) , consistent with Section 2.3. This enables direct visualization of the InEKF uncertainty as a projected hyperellipsoid, which is particularly useful as such filters are increasingly adopted in robotics.

**Use of AI:** The plotting code and the comparison scripts for SciPy, spatialmath, and manifpy (available at https://github.com/AlexanderFabisch/pytransform3d-frontiers-rai/) were developed with the assistance of Claude (Claude-sonnet-4-6, Anthropic, https://claude.ai). In particular, we used Claude to ensure that we use all libraries as effectively as possible in terms of code length efficiency and execution time by scanning the documentation and source code.

#### 3.3.1 Relevant code for plotting

As an example, we selected trajectory F16 from the Vulcano Island dataset, where the robot moves in a straight line over rocky terrain for a distance of 25 m. Figure 10 shows the InEKF state estimation and GNSS-based positions. As GNSS does not provide orientation data, we applied the Kabsch transformation to the first 2 m of the trajectory to obtain the optimal yaw angle and align the initial robot world frame with the GNSS frame. Listing 4 exemplifies the following parts of the code for this example: (1) loading the trajectory data from a CSV file and the robot URDF; (2) aligning the ground truth to the robot world frame, which involves batch conversion and concatenation of poses; and (3) displaying the covariance hyperellipsoids. A zoomed view in Figure 10 shows the robot model based on joint encoder data and the InEKF orientation at selected time steps together with the uncertainty Σ , visualized as a projected equiprobable hyperellipsoid. Uncertainty and position drift increase indefinitely over time due to cumulative odometry errors and the lack of external observations from drift-free sensors, such as a camera or lidar-based map localization.

**FIGURE 10:**
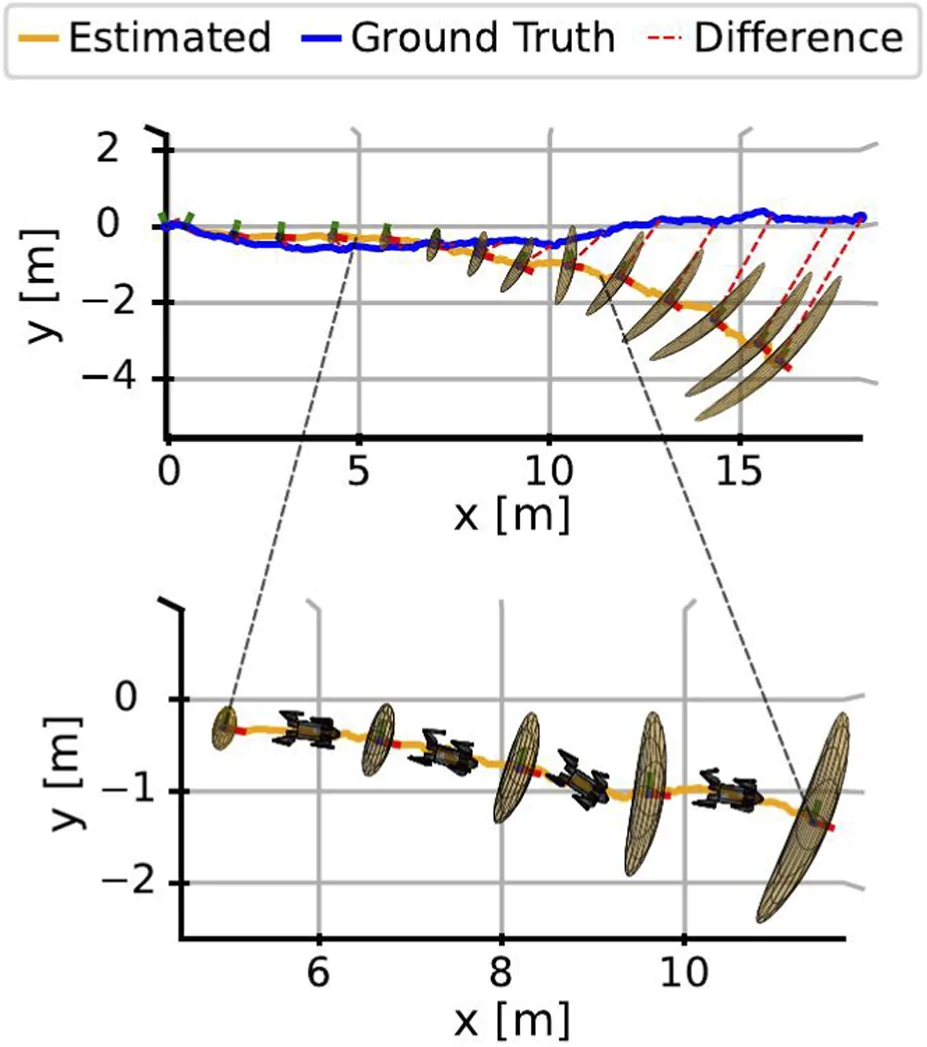
Illustration of an estimated robot pose versus ground truth from the Vulcano Island field test dataset (Popescu et al., 2026b). We highlight the differences between Cartesian positions at discrete steps in time with a dashed red line. The estimated covariance of the pose is displayed as a 3σ 6D hyperellipsoid (corresponds to 99.7% probability) that is projected to the 3D Cartesian space. Below the overview plot, we zoom in to focus on a part of the trajectory. In this plot, we alternate between showing the hyperellipsoid and robot state including joint configurations from the log data and mean of the estimated pose. Both plots are the top views of 3D plots in Matplotlib. ☛ Code.

Listing 4 Quadruped state estimation. ☛ Code.

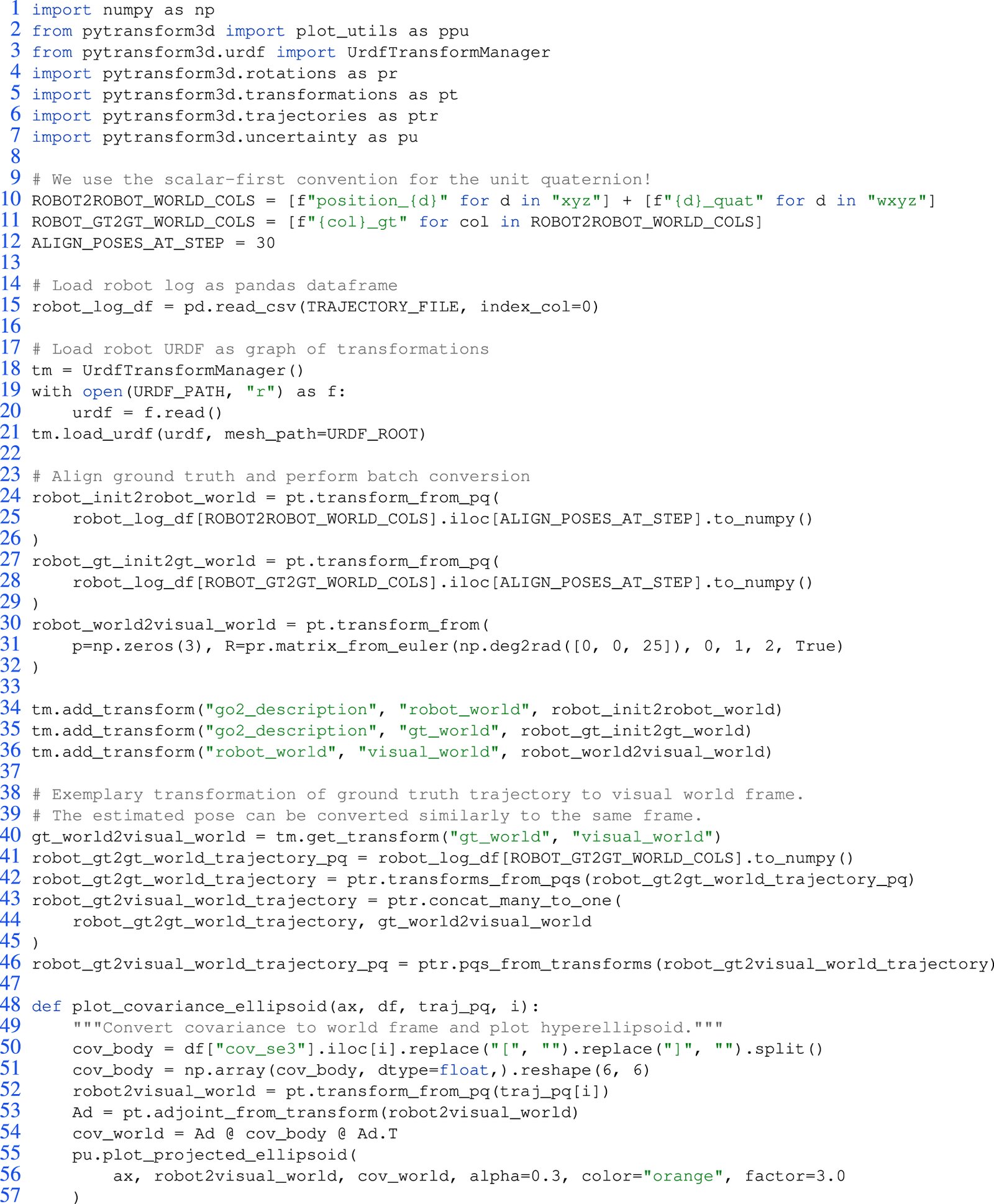

#### 3.3.2 Comparison for trajectory alignment (batch operations)

Listing 5 compares the trajectory alignment between pytransform3d and manif. Both scripts solve the same task: computing a world-frame alignment transform from a reference pose and converting the full trajectory to a common visualization frame, yielding a 2D NumPy array of position and quaternion per step. Both code examples were formatted with black and with the maximum line length set to 100. Both use similar variable names. The code for pytransform3d is slightly shorter (28 vs. 34 lines) even though it has long, explicit function names. Composition operations with pytransform3d are easily verifiable by checking the order of successive frame names. Furthermore, it is less complex, as it implements vectorized operations and does not require list comprehensions. manif is an example of a library that uses an object-oriented approach and does not support vectorization of the required conversion and composition operations. Similarly, spatialmath does not allow vectorized conversion from position and quaternion to its SE3 class, and SciPy does not have a function that constructs RigidTransform directly from a NumPy array with seven elements that represent position and an orientation quaternion. Among the libraries SciPy, spatialmath, manif, and pytransform3d, pytransform3d is the fastest as it vectorizes all operations with NumPy, although SciPy uses Cython (Behnel et al., 2011) to compile its code; manif is implemented in C++. We measured computation times over 20 repeated runs on a single CPU core. pytransform3d achieves a mean of 3.1 ms, compared with 7.1 ms for SciPy, 7.4 ms for manif, and 107.0 ms for spatialmath. The large gap to spatialmath is due to its lack of vectorized conversion from position–quaternion arrays to SE3, forcing a Python loop over all trajectory poses.

Listing 5 Trajectory alignment with pytransform3dand manif. ☛ Code.

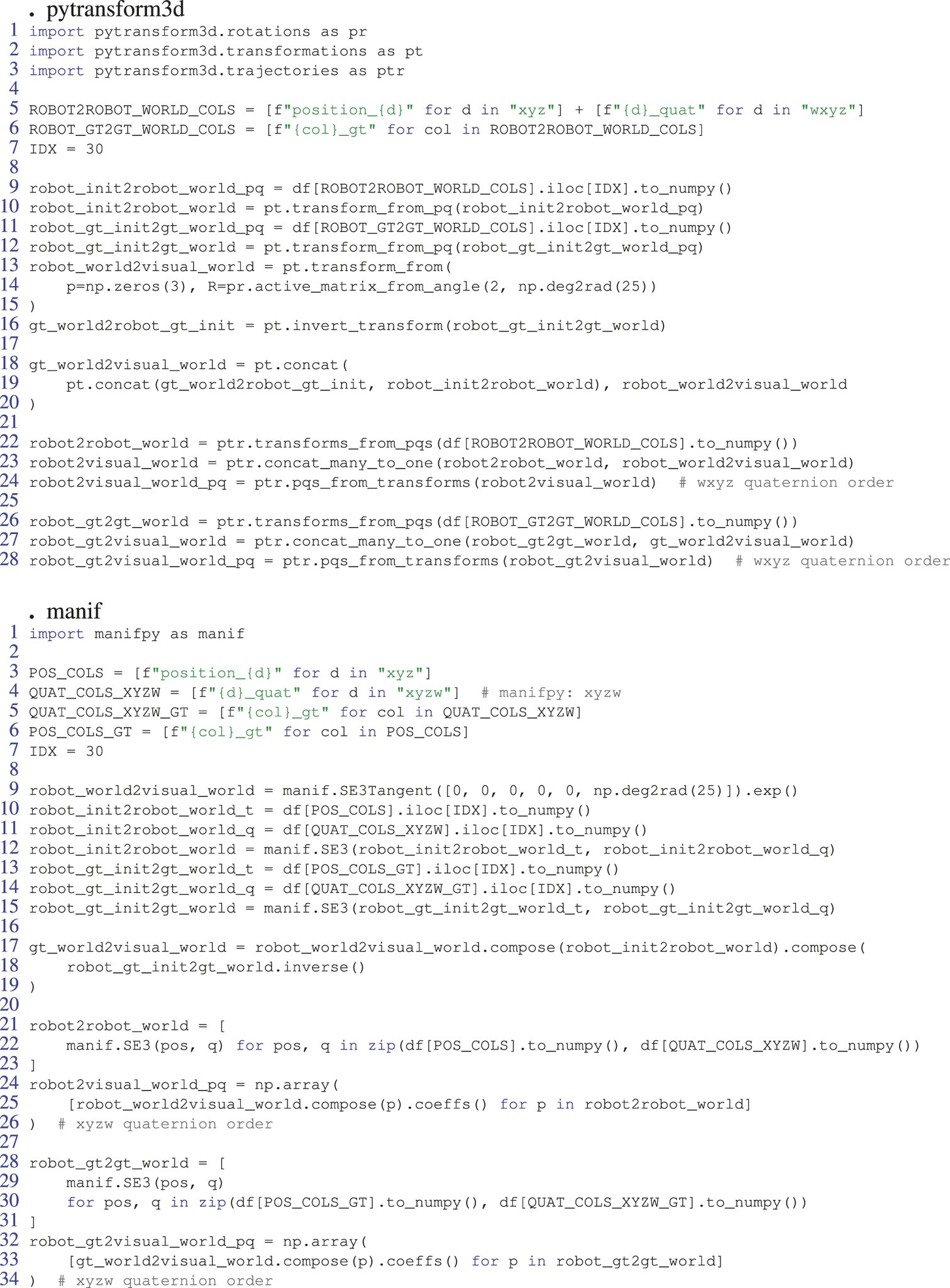

## 4 Discussion

### 4.1 Summary

We introduce common representations of 3D transformations and show how they are implemented in pytransform3d along with tools such as TransformManager, visualization tools, and uncertainty-handling methods. We demonstrate that TransformManager can be used to implement an automatic embodiment mapping, the uncertainty module simplifies the implementation of uncertainty propagation for state estimation and probabilistic product of exponentials, and the visualization and plotting tools can be used for debugging and to produce publication-ready figures. With pytransform3d, we provide a lightweight tool that helps roboticists deal with rigid transformation-related problems. We show that it can be applied specifically in complex kinematic problems that require handling of graphs of transformations or uncertainty and for batch processing of transformations, visualization, and plotting.

### 4.2 Limitations

pytransform3d lacks real-time capability in comparison with more efficient implementations. ROS tf (Foote, 2013) or similar systems that are implemented in C++ are more suitable in this case. The recommended way of implementing real-time applications with pytransform3d is to translate the well-readable source code of the required functions of the library into a faster programming language, such as C, C++, or Rust. Furthermore, the library only focuses on kinematics, not on the dynamics of robotic mechanisms.

### 4.3 Future work

Some of the core functionality of the library could be moved to general purpose libraries such as SciPy or Matplotlib. This already started with the integration of conversions from and to dual quaternions and exponential coordinates of transformation in the RigidTransform class of SciPy (Shambaugh, 2025).

For applications in machine learning and differentiable programming, the core functionality of the library can be implemented with a library for automatic differentiation. An implementation based on JAX (Bradbury et al., 2018) called jaxtransform3d with features similar to PyTorch3D (Ravi et al., 2020) is currently under development (Fabisch, 2025a). jaxtransform3d is inherently vectorized, GPU-accelerated, and fully differentiable. However, it is still in an experimental development stage.

## Data Availability

The original contributions presented in the study are included in the article/supplementary material, further inquiries can be directed to the corresponding author.
